# Pituitary Apoplexy in Macroadenoma After Minor Surgery: An Unusual Case and Literature Review

**DOI:** 10.7759/cureus.57912

**Published:** 2024-04-09

**Authors:** Melanie Buchta, Herbert Krainz, Jochen Grimm, Theo Kraus, Christoph J. Griessenauer, Christoph Schwartz, Moritz F Ueberschaer, Martin Dejaco, Ferdinand Otto

**Affiliations:** 1 Neurosurgery, Christian Doppler Medical Center, Paracelsus Private Medical University, Salzburg, AUT; 2 Neuroradiology, Christian Doppler Medical Center, Paracelsus Private Medical University, Salzburg, AUT; 3 Pathology, University Hospital Salzburg, Paracelsus Private Medical University, Salzburg, AUT; 4 Otolaryngology - Head and Neck Surgery, University Hospital Salzburg, Paracelsus Private Medical University, Salzburg, AUT; 5 Neurology, Christian Doppler Medical Center, Paracelsus Private Medical University, Salzburg, AUT; 6 Neurology, Centre for Cognitive Neuroscience, Salzburg, AUT

**Keywords:** case report, ocular palsy, transsphenoidal resection, pituitary adenoma, postoperative pituitary apoplexy

## Abstract

Pituitary apoplexy is a rare and severe complication of pituitary adenoma that may present with new-onset headache, ocular palsy, visual disturbances, life-threatening electrolyte imbalance, and endocrinological disturbances due to pituitary hemorrhage and/or infarction.

We report the case of a 58-year-old previously healthy patient who developed isolated mild oculomotor nerve palsy of the left eye following osteosynthesis of a traumatic right distal radius fracture. Initial cerebral magnetic resonance imaging showed a pituitary macroadenoma without characteristic signs of pituitary infarction or hemorrhage. The patient presented to the neurology department on the fifth postoperative day with malaise and fatigue due to pituitary insufficiency, deteriorated rapidly and required intensive care monitoring. Clinical stabilization was achieved through the administration of hydrocortisone, and transsphenoidal resection of the pituitary lesion was performed on the 10th day after acute symptom onset. Histological examination revealed a necrotic pituitary adenoma.

Pituitary apoplexy may occur after minor surgery in patients with pituitary adenoma. Clinicians should pay particular attention to laboratory signs of pituitary insufficiency in new-onset oculomotor nerve palsy associated with sellar lesions, as cerebral imaging may miss pituitary apoplexy and therefore delay diagnosis and treatment. In our case, delayed decompressive transsphenoidal resection resulted in the normalization of the oculomotor nerve palsy while the pituitary insufficiency persisted. The potential impact of an earlier surgical intervention on the outcome of pituitary function remains uncertain.

## Introduction

Pituitary apoplexy (PA) is a rare and severe complication of pituitary adenomas, mostly caused by acute/subacute hemorrhage or infarction. Of all patients with pituitary adenoma, 0.6-10% will develop clinically significant pituitary apoplexy [1,2]. In most cases, the existence of a pituitary tumor is unknown at the time of apoplexy [3,4]. PA may present with one or more of the following clinical signs and symptoms: ocular palsy, visual loss, severe headache, nausea, vomiting, electrolyte imbalance, and endocrine abnormalities [4-6]. The pathogenesis of PA is not fully understood [7] and associated risk factors include large tumor size [8], dynamic pituitary testing and hormonal treatment [9], head trauma, and surgery [3]. Several cases of PA have been reported after endovascular treatment, laparoscopic surgery [3,10], cardiac surgery [11], and orthopedic surgery [3,12]. Cranial magnetic resonance imaging (MRI) is the imaging modality of choice to detect PA [13]. We report a case of PA after minor surgery with subacute symptom-onset and delayed diagnosis due to inconclusive imaging.

## Case presentation

**Figure 1:**
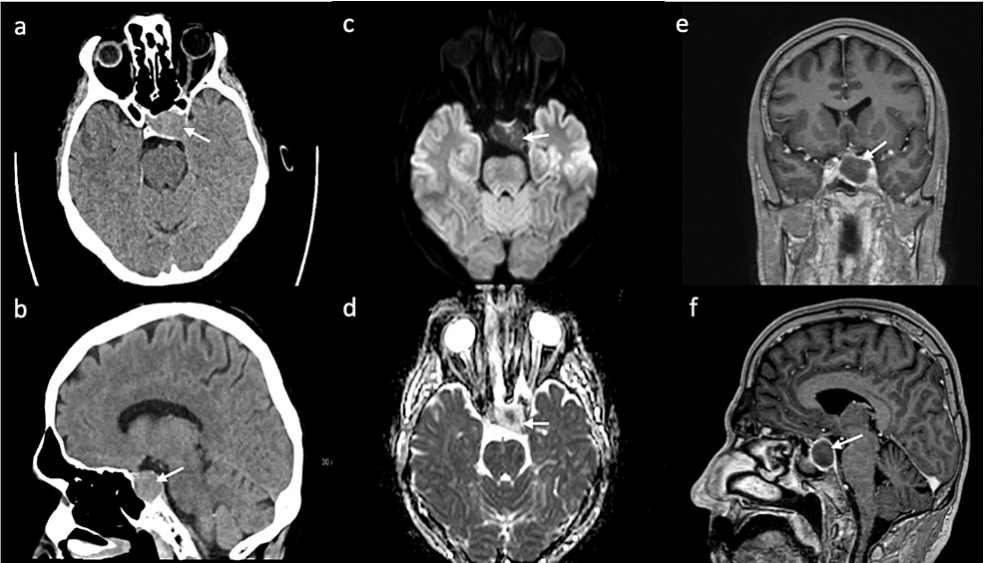
Initial unenhanced CT showing a sellar mass (arrow) with cavernous sinus infiltration of intermediate density in axial (a) and sagittal (b) images. MRI was performed two days later. Diffusion-weighted axial images show a central area of low ADC (d) but otherwise no diffusion restriction in the mass (c). Contrast enhanced T1-weighted images show marginal enhancement of the mass in the coronal (e) and sagittal (f) images. CT: computed tomography, MRI: magnetic resonance imaging, ADC: apparent diffusion coefficient.

A 58-year-old previously healthy man was hospitalized for surgical treatment of a right distal radius fracture sustained in a bicycle accident. After the surgical treatment of the fracture, the patient noticed double vision and a drooping of the left eyelid. An initial cranial computed tomography (CCT) scan revealed a 2.8 x 1.9 x 2 cm soft-tissue dense mass of the pituitary gland. Subsequent cranial MRI confirmed a pituitary lesion with peripheral ring enhancement on contrast-enhanced imaging without definite evidence of hemorrhage and/or infarction (Figure 1). Neurologic examination revealed moderate ptosis, reactive pupils, mild palsy of elevation, depression, and adduction of the left eye, with no evidence of other deficits. In addition, the patient reported a history of diffuse headaches for approximately one year. 

**Figure 2:**
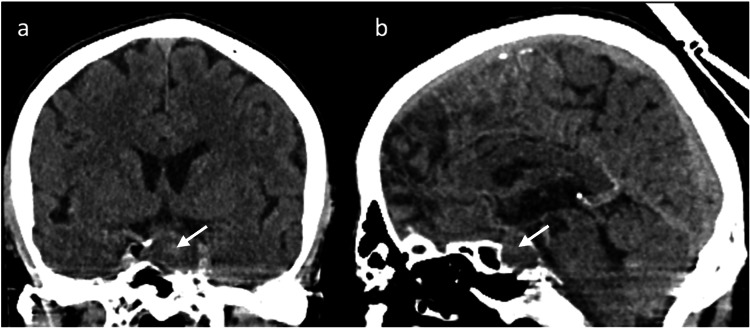
Follow-up CT five days after the initial CT shows coronal (a) and sagittal (b) images with progressive hypodensity of the sellar mass with some central hyperdensity (arrow). CT: computed tomography.

**Figure 3:**
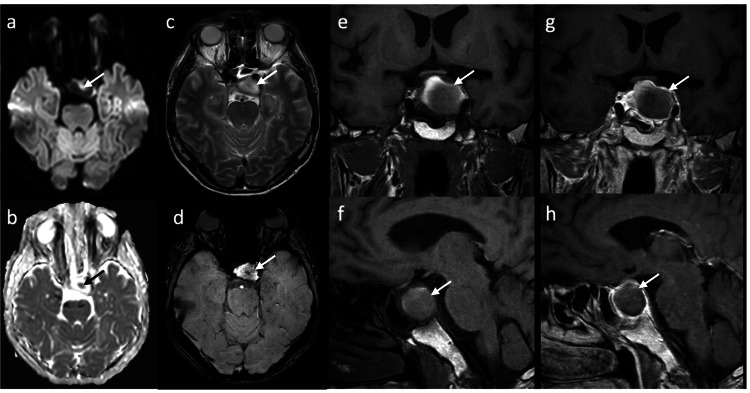
Follow-up MRI eight days after initial CT shows a central area of diffusion restriction (arrow) on axial diffusion-weighted images (a and b). The mass (arrow) is relatively hyperintense on axial T2-weighted images (c) and shows a central susceptibility artefact compatible with hemorrhage or infarction on axial susceptibility-weighted images (d). T1-weighted coronal (e) and sagittal (f) images show incomplete peripheral hyperintensity. After contrast administration, the peripheral enhancement appears slightly less pronounced on coronal (g) and sagittal (h) T1-weighted images than on the initial MRI. CT: computed tomography, MRI: magnetic resonance imaging.

During hospitalization, the patient developed dizziness, apathy, nausea, and vomiting on the fifth postoperative day. At that time, a neurologic examination revealed complete left oculomotor nerve palsy. Laboratory tests listed in Table 1 showed marked hyponatremia, hypoglycemia, and pan-hypopituitarism, including low levels of thyroid-stimulating hormone (TSH), testosterone, corticotropin, and cortisol. Follow-up CCT showed a central hyperdensity of the pituitary lesion (Figure 2). MRI showed hyperintensity on the T2-weighted image and hypointensity on the T1-weighted image with a central susceptibility artifact compatible with acute infarction of the pituitary lesion with a focal hemorrhagic component (Figure 3). 

Substitution therapy with hydrocortisone (20 mg/d) was initiated, and transsphenoidal surgery was performed after normalization of electrolytes and blood glucose levels on the 10th day after the acute onset of pituitary insufficiency.

**Table 1:** Patients' laboratory test results after acute onset of symptoms on postoperative day 5 following right distal radius osteosynthesis. TSH: Thyroid-stimulating hormone; FT4: Free thyroxine; FT3: Free triiodothyronine; FSH: follicle stimulating hormone; LH: luteinising hormone; ACTH: adrenocorticotropic hormone. mmol/l = millimol per litre; mg/dl = milligram per deciliter; mU/l = milliunit per litre; ng/dl = nanogram per deciliter; pmol/l = picomol per litre; µU/ml = microunit per millilitre; ng/ml = nanogram per millilitre.

Lab tests	Result	Normal range
Sodium	112 mmol/l	135 - 148 mmol/l
Glucose	53 mg/dl	70 - 100 mg/dl
TSH	0.06 mU/l	0.50 - 4.20 mU/l
FT4	1.01 ng/dl	0.93 - 1.70 ng/dl
FT3	1.3 pmol/l	3.1 - 6.8 pmol/l
Prolactin	156 µU/ml	86.0 - 324.0 µU/ml
FSH	1.8 mU/ml	1.5 - 12.4 mU/ml
LH	0.3 mU/ml	1.7 - 8.6 mU/ml
ACTH	< 1.5 pg/ml	7.2 - 63.3 pg/ml
Cortisol	13.60 ng/ml	26.80 - 184.00 ng/ml
Testosterone	< 0.02 ng/ml	1.93 - 7.40 ng/ml

**Figure 4:**
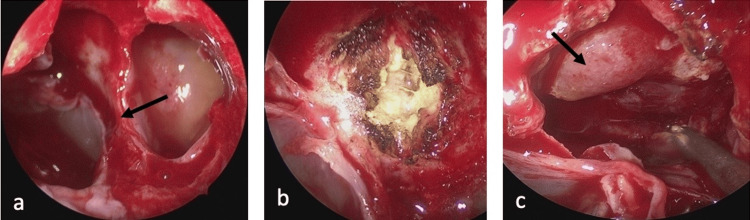
Intraoperative images. Both sides of the sphenoid sinus with the sphenoid septum (black arrow) are shown before mucosal excision (a). The mucosa is removed and the lesion is located on the posterior wall of the sphenoid sinus. The lesion is shown after coagulation and incision (b). After partial removal of the pathological tissue, some vital looking pituitary tissue is visible (black arrow) (c).

Endoscopic endonasal transsphenoidal resection of the lesion in the sella turcica was performed by the otolaryngology and neurosurgery team using a CT- and MRI-fused navigation system (Brainlab AG, Munich, Germany). A binostril approach with a wide sphenoidal opening was performed in a standardized manner (Figure 4a). By further resection of the mucosa and posterior wall of the sphenoid sinus, the lesion was identified and centrally coagulated with bipolar cautery and sharply opened in an "X" pattern with a scalpel (Figure 4b). Homogeneous, yellowish, and necrotic-appearing tissue was then removed with a ring curet and suction. The frozen section was consistent with pathologic pituitary tissue. The pituitary gland was identified and preserved at the diaphragma sellae on the right side (Figure 4c). Endoscopic inspection revealed some pathologic tissue remainder in proximity to the internal carotid artery on the right side, which was not removed due to the high-risk location. The resection cavity was packed with gel foam, lined with fibrin glue, and covered with the previously removed mucosa to provide a watertight closure.

Postoperatively, the patient did not report headache, confusion, dizziness, nasal congestion, rhinoliquorrhea, or other new focal neurologic deficits. The application of desmopressin led to regression of the postoperative diabetes insipidus. Cerebral MRI on the first postoperative day showed a residual lesion near the right carotid artery, but no evidence of acute hemorrhage or ischemia. Postoperative visual field testing was normal, and the motility of the left eye, mydriasis (light reaction 5.2 mm left eye, 4.8 mm right eye), and ptosis (9-10 mm) improved significantly. Hormonal replacement was adjusted postoperatively. Thus, hydrocortisone was reduced to a maximum daily dosage of 30 mg, and L-thyroxine therapy was increased to 100 µg per day. At the two-month follow-up, the patient was started on testosterone replacement therapy for hypogonadotropic hypogonadism. Serum electrolytes remained within normal range limits and the patient had normal ocular motility. 

**Figure 5:**
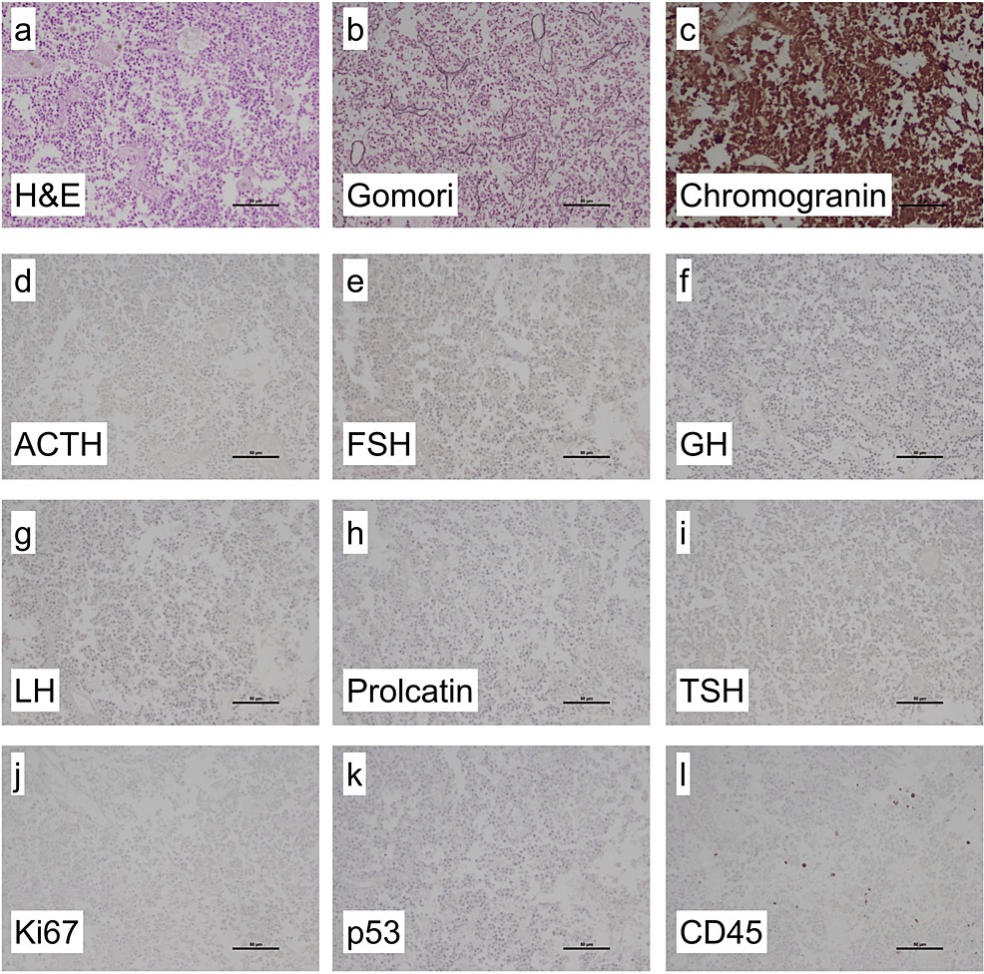
Histological findings. H&E stained sections showed necrotic pituitary tissue without lobules (a). Gomori silver stain confirmed loss of lobular structure (b). Immunohistochemical reactions with antibodies against Chromogranin showed neuroendocrine origin (c). Immunohistochemistry with antibodies against pituitary hormones showed no expression of ACTH (d), FSH (e), GH (f), LH (g), prolactin (h) and TSH (i). The proliferation index using Ki67 antibody was <1% (j). There was no aberrant p53 expression (k). Antibodies against CD45 showed some leukocytes (l). TSH: Thyroid-stimulating hormone, GH: growth hormone, FSH: follicle-stimulating hormone, LH: luteinizing hormone, ACTH: adrenocorticotropic hormone

Neuropathologic analysis of the obtained tissue showed necrotic pituitary tissue with loss of lobular structure in H&E stain. Gomori silver stain confirmed loss of lobules. Immunohistochemical reactions with antibodies against Chromogranin showed neuroendocrine origin. Additional immunohistochemistry of pituitary hormones showed loss of expression of adrenocorticotropic hormone (ACTH), follicle-stimulating hormone (FSH), growth hormone (GH), luteinizing hormone (LH), prolactin and TSH. Proliferation was low with less than 1% of Ki67-positive cells. No p53 expression was detected. Using antibodies against CD45, leucocytes were detected. Integrating histology and immunohistochemistry showed necrotic pituitary tissue without lobules and without hormone expression, in line with necrotic pituitary adenoma (Figure 5).

## Discussion

PA is an uncommon and potentially life-threatening clinical syndrome in patients with pituitary adenoma that may occur after minor surgery. We describe a patient who developed PA after osteosynthesis of the right distal radius, initially presenting with mild symptoms and inconclusive imaging showing a sellar mass without clear evidence of a pituitary hemorrhage or infarction, leading to a delayed diagnosis and treatment. 

The precipitating factors for PA are not fully understood. It has been hypothesized that surgical procedures may cause fluctuations in blood flow and blood pressure and the development of microemboli in the setting of impaired hemostasis, leading to infarction and hemorrhage [3,14]. A study by Kruse et al. recorded the intrasellar pressure of patients with pituitary adenoma during surgery and showed that pituitary adenoma has a critical perfusion pressure, which may lead to PA due to a decrease in arterial blood pressure [15]. Several cases of postoperative PA have been reported after orthopedic surgery, such as posterior lumbar fusion [12], hip replacement [3], laparoscopic surgery [10], or cardiac surgery [11]. These surgeries are often associated with blood pressure fluctuations and intraoperative anticoagulant therapy in the case of cardiac surgery or transient increases in intracranial pressure during laparoscopic surgery [10,11]. In our case, systolic blood pressure fluctuated between 75 and 145 mmHg during the surgery, which may have caused insufficient blood supply and subsequent ischemia of the present pituitary macroadenoma. 

Cerebral imaging remains a challenge in patients with sellar lesions, particularly in the setting of suspected PA. CCT is commonly used as the first emergency imaging modality in patients with sudden onset of new focal neurological deficits, including symptoms associated with possible PA. While CCT is able to detect a pituitary tumor in approximately 90% of the cases, pituitary hemorrhage is detectable in only 20-40% of the cases [13,16]. Therefore, the imaging modality of choice in patients with suspected PA is MRI, which detects a hemorrhagic transformation in up to 89% of the cases [13]. In the acute phase, blood is isointense on T1-weighted images and isointense to hypointense on T2-weighted images. PA may appear with different MRI characteristics, including different stages of hemorrhage and non-hemorrhagic components [17]. In our case, MRI showed a nonspecific, slightly heterogeneous signal on T1- and T2-weighted images with a very small hemorrhagic component that was only detectable on susceptibility-weighted imaging (SWI) in the follow-up MRI (Figure 3). Peripheral ring enhancement was visible on MRI and appeared less intense on follow-up MRI (Figure 3); this has been described on contrast-enhanced MRIs in cases of PA [11,18], but can also be seen in cystic adenoma and craniopharyngioma and is therefore not specific [16,19]. An area of infarction typically shows a high signal on diffusion-weighted imaging (DWI) with a corresponding low apparent diffusion coefficient (ADC) as seen in PA [19]. In our patient, no diffusion restriction was seen on the initial MRI, whereas on the follow-up MRI on the fifth day after symptom onset, DWI showed a high signal in a small area with corresponding low ADC in the center of the tumor mass, consistent with hemorrhage or infarction, but no generalized diffusion restriction.

Of note, the initial postoperative MRI showed no evidence of PA despite the presence of mild oculomotor nerve palsy. After an acute onset of symptoms, a subsequent MRI confirmed the presence of PA. The underlying explanation for this clinical course may be a gradual and progressive hemorrhagic event accompanied by a compressive effect leading to PA of the macroadenoma. 

Once PA is diagnosed, treatment should be initiated rapidly to prevent life-threatening conditions and permanent neurological deficits. We initially focused on conservative therapy of pituitary insufficiency, consisting of hormone replacement therapy and stabilization of electrolyte abnormalities. Delayed endoscopic transsphenoidal resection was performed 10 days after the onset of acute symptoms. At the two-month follow-up, the patient had normal ocular motility, and hormone replacement therapy with low-dose prednisone, L-thyroxine, and testosterone was established due to residual panhypopituitarism. In the absence of randomized prospective trials, the optimal management of PA remains unclear. There are several studies and meta-analyses with variable results according to the type of intervention, conservative management versus surgery, outcome, and timing of surgery [3,7,20]. Patients selected for conservative management mostly had milder neuro-ophthalmic deficits or improving symptoms, whereas surgically managed patients had a more severe clinical presentation [3]. Whether there is a difference in outcomes between these treatment groups remains inconclusive in most of the analyzed studies [3,7]. Several studies have reported improvement in visual deficits and cranial nerve palsies, and less improvement in pituitary function, regardless of the timing of surgery [3]. It should be noted that the definition of early versus delayed timing was variable between studies and surgery was performed after electrolyte and hemodynamic stabilization [7]. 

## Conclusions

PA is a life-threatening complication of pituitary adenoma which may lead to endocrine failure, severe electrolyte imbalance, and diminished level of consciousness. PA imaging can be ambiguous and difficult to interpret, leading to delayed diagnosis and therapeutic management. Pituitary insufficiency and electrolyte imbalances should be screened in a patient presenting with sudden postoperative oculomotor palsy with a newly diagnosed sellar mass. In cases of suspected PA or incidental pituitary mass, MRI protocols should include DWI and SWI sequences. In our particular case, delayed decompressive transsphenoidal resection led to normalization of the oculomotor nerve palsy while the pituitary insufficiency persisted. The possible influence of an earlier surgical intervention on the outcome of pituitary function remains uncertain.
